# White piedra: fungal extracellular matrix formation and its importance in pathogenesis. An ultrastructural study^☆^

**DOI:** 10.1016/j.abd.2025.501140

**Published:** 2025-07-03

**Authors:** Hiram Larangeira de Almeida Jr., Thales de Moura Assis, Eduardo Camargo Faria, Viviane Mazo Fávero Gimenes

**Affiliations:** a Postgraduate Studies in Health and Behavior, Department of Dermatology, Universidade Federal de Pelotas, Pelotas, RS, Brazil; b Postgraduate Studies in Health and Behavior, Universidade Católica de Pelotas, Pelotas, RS, Brazil; c Instituto de Medicina Tropical de São Paulo, Laboratório de Micologia (LIM53), São Paulo, SP, Brazil

**Keywords:** Extracellular matrix, Scanning electron microscopy, White piedra

## Abstract

**Background:**

White piedra is a disease caused by some species of the genus *Trichosporon*. A case of white piedra was investigated, whose molecular examination identified *Cutaneotrichosporon* (*Trichosporon) debeurmannianum* as the causative agent.

**Methods:**

Scanning electron microscopy (SEM) was used to examine the affected hairs, as well as the fungal colony of *C. debeurmannianum* obtained from the hairs. For comparative purposes, a colony of *Trichosporon mucoides* obtained from a mycotheque was also examined.

**Results:**

Examination of the affected hairs using SEM easily demonstrates nodules on the hair shaft with a predominance of rounded yeast-like structures, adhered to each other by a cementing substance. Examination of the *C. debeurmannianum* colony demonstrates significant adhesion between the fungal cells by a reticular extracellular matrix. Examination of the *T. mucoides* colony obtained from a mycotheque demonstrates a small production of fibrillar substance between the blastoconidia.

**Discussion:**

Examination of the colony obtained from the piedra showed significant formation of extracellular matrix, adhering to and covering the fungal structures, forming a biofilm. This matrix must correspond to the cementing substance described in the condition.

**Conclusion:**

The synthesis of the extracellular matrix must be crucial in the formation of white piedra nodules.

## Introduction

The genus *Trichosporon* comprises yeast-like basidiomycete fungi (forming blastoconidia and hyphae),^1^, ^2^, ^3^ with more than 50 described species, which cause dermatoses and opportunistic infections.

The genus received this name (*Trichos*-hair and *sporon*-spores) because it was first described from white piedra nodules by Beigel in 1865, and was later named *Trichosporon beigelii*.^1^, ^2^ Currently, the main causes of disease in humans, according to the new taxonomy, are: *T. asahii*, *T. asteroides*, *T. cutaneum*, *T. mucoides*, *T. inkin* and *T. ovoides*.^1^

Fungi of this genus cause superficial mycoses such as white piedra and onychomycosis, with the latter being usually caused by *T. cutaneum*, but they also cause systemic infection, being the second cause of fungal septicemia in hematologic oncology patients, with a frequency lower only than that of the genus *Candida spp*.^1^

White piedra is a condition generally caused by some species of the genus *Trichosporon*, such as *T. cutaneum*, *T. inkin*, *T. ovoides*, and *T. loubieri*. It is characterized by small nodules that form on the hair shaft and can affect various regions of the body, such as the beard, scalp, pubic hair, armpits, and eyebrows. Long hair could facilitate its occurrence and it is more common in children.^3^, ^4^, ^5^

This genus has undergone numerous reclassifications in recent years.^6^, ^7^ The fungus *Cutaneotrichosporon* (*Trichosporon*) *debeurmannianum*, also called *Trichosporon debeurmannianum* by some authors, was identified in 2001,^8^ and has already been described as causing skin infection.^9^, ^10^ A case of white piedra and the respective fungal culture were assessed, whose molecular examination identified *C. debeurmannianum* as the cause of the condition.

## Methods

Hair affected by a case of white piedra was examined using Scanning Electron Microscopy (SEM) with a Jeol microscope, JSM-6610LV at Centro de Microscopia do Sul - CEMESUL - FURG, as well as the fungal colony obtained from the hair culture on Sabouraud dextrose agar. For comparative purposes, a colony of *Trichosporon mucoides*, obtained from the mycotheque of Instituto de Medicina Tropical de São Paulo, was also examined.

The hair was examined after gold metallization. To examine the colonies, a small section of the culture obtained was removed, fixed in glutaraldehyde, dehydrated and subsequently submitted to gold metallization.

## Results

**Figure 1:**
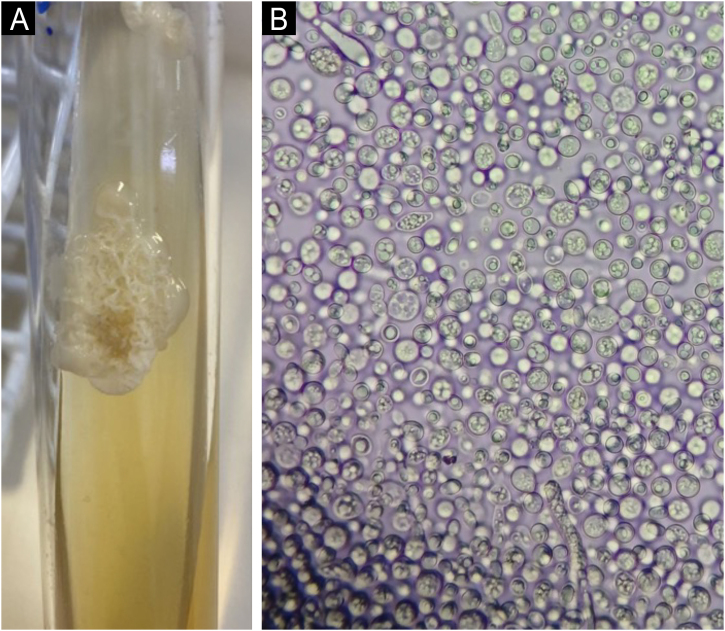
(A) Colony with creamy aspect. (B) Optical microscopy – predominance of blastoconidia with some hyphae.

The colony obtained from the affected hairs showed a typical yellowish-creamy appearance (Fig. 1A). Examination of the culture with optical microscopy revealed yeast-like structures (blastoconidia) and some filaments (Fig. 1B).

**Figure 2:**
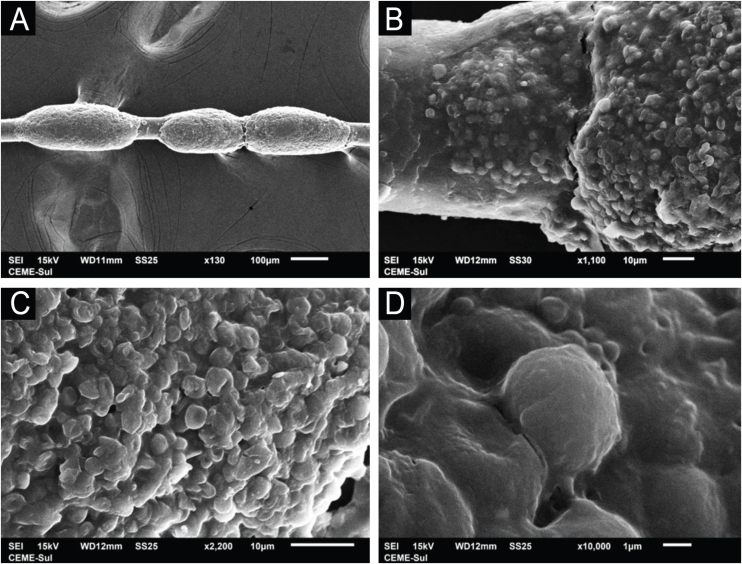
SEM of white piedra ‒ (A) Low magnification identifying nodules in the hair (×130). (B) Detail of the nodule, with initial lesion on the left (×1,100). (C) High magnification demonstrating clusters of blastoconidia (×2,200). (D) High magnification with blastoconidium on the surface (×10,000).

Examination of the affected hairs with SEM easily demonstrates the nodules at low magnification (Fig. 2A). At high magnification, well-formed nodules, and incipient lesions are observed (Fig. 2B). Detailed examination of the nodules shows their formation with a predominance of rounded yeast-like structures, similar to the findings of optical microscopy, adhered to each other by a cementing substance (Fig. 2C‒D).

**Figure 3:**
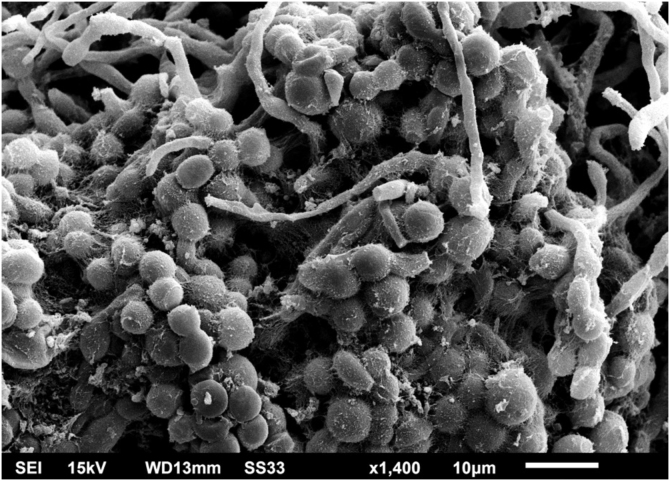
SEM of the *C. debeurmannianum* colony with a predominance of blastoconidia and some hyphae (×1.400).

**Figure 4:**
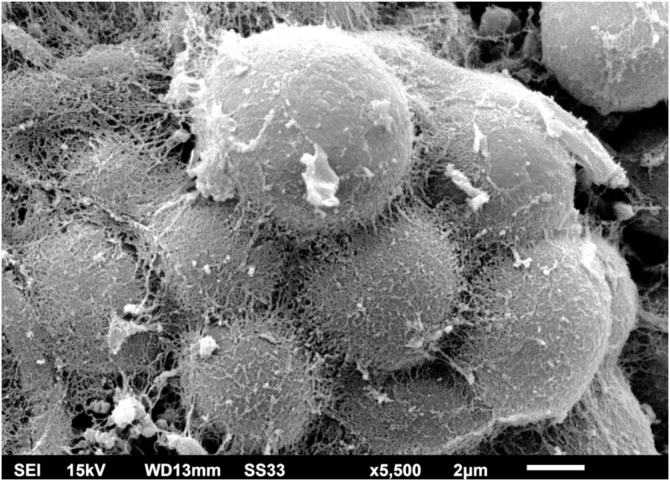
SEM of the *C. debeurmannianum* colony – detail of the blastoconidia adhered by reticular extracellular matrix, forming clusters resembling acini (×5.500).

**Figure 5:**
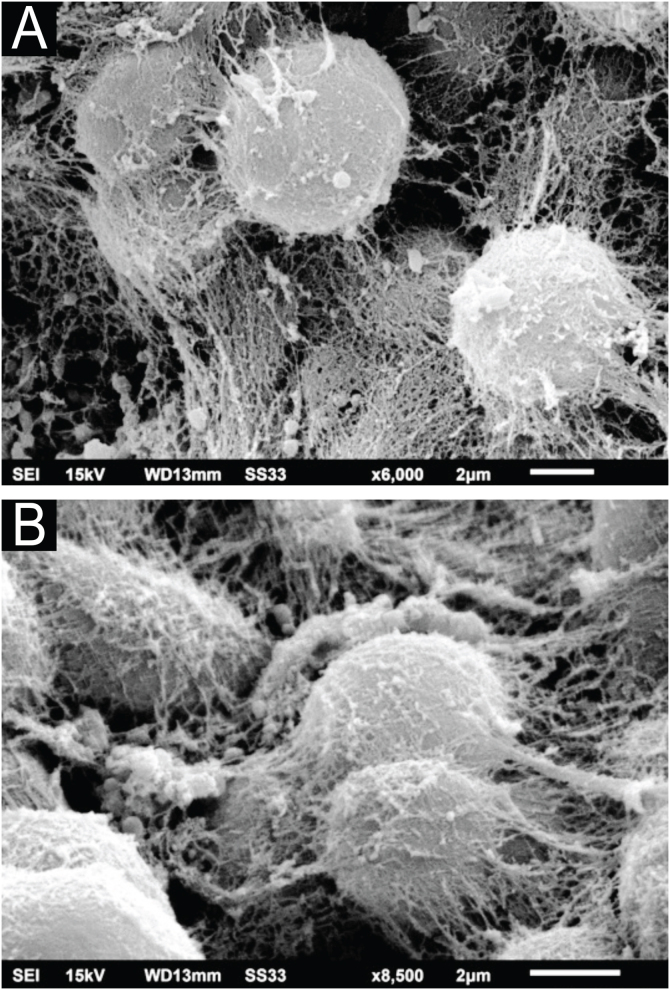
SEM of *C. debeurmannianum* colony ‒ (A) High magnification of blastoconidia covered by extracellular matrix (×6,000). (B) Detail of covered blastoconidia and adhered to each other (×8.500).

**Figure 6:**
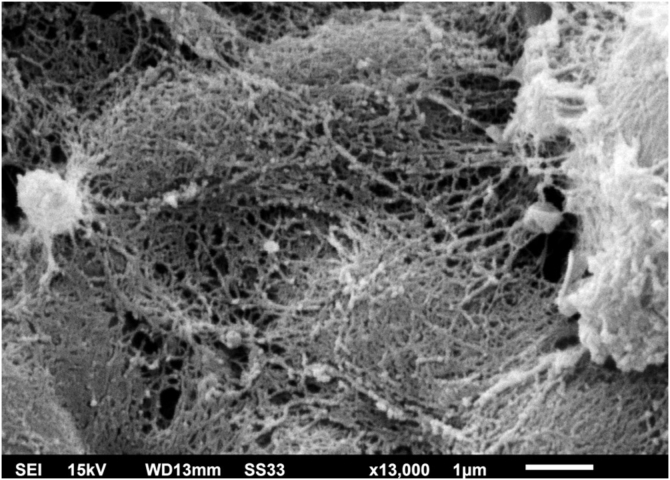
SEM of *C. debeurmannianum* colony – detail of dense extracellular matrix covering the blastoconidia (×13.000).

**Figure 7:**
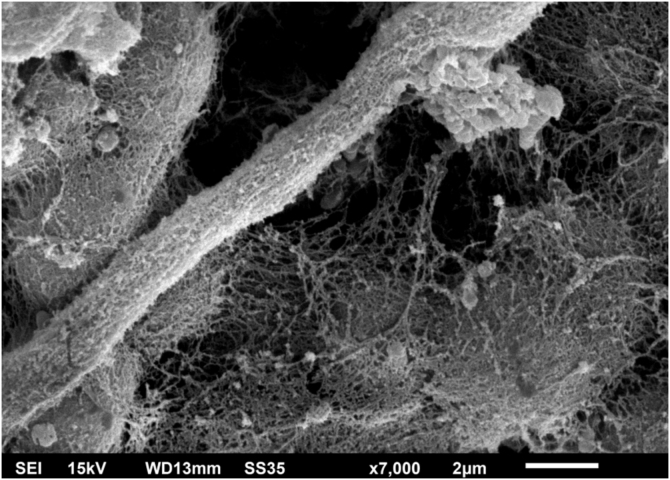
SEM of the *C. debeurmannianum* colony – detail of the extracellular matrix formation also by hyphae (×7.000).

Examination of the *C. debeurmannianum* colony with SEM also demonstrates a predominance of rounded structures, as seen under optical microscopy, and at low magnification, adhesion between these structures by an extracellular network is observed; the hyphae show a lower production of this network (Fig. 3). Detailed examination shows significant adhesion between the rounded cells, sometimes forming clusters that resemble “acini” (Fig. 4); the blastoconidia appear well surrounded by the extracellular matrix (Fig. 5). In some fields, this extracellular network hides the yeast-like structures, compacting them in this matrix (Fig. 6). Some hyphae were also seen with this extracellular matrix (Fig. 7).

**Figure 8:**
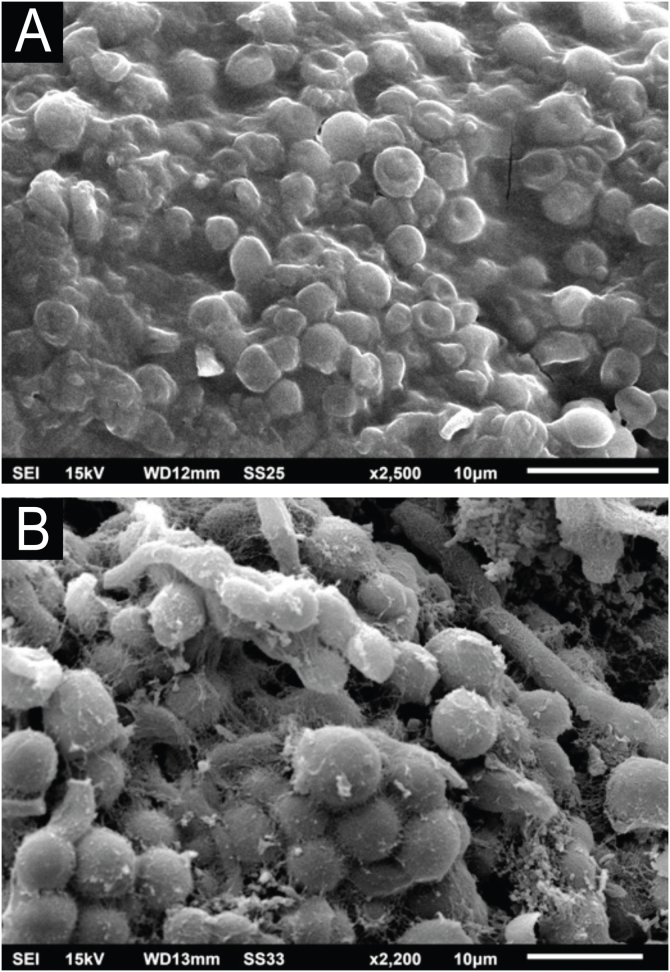
SEM – comparative demonstration of the similarity between the appearance of the white piedra nodule (A; ×2,500) and the *C. debeurmannianum* colony (B; ×2.200).

A comparison at similar magnifications shows the similarity between the white piedra nodule and the *C. debeurmannianum* culture (Fig. 8).

**Figure 9:**
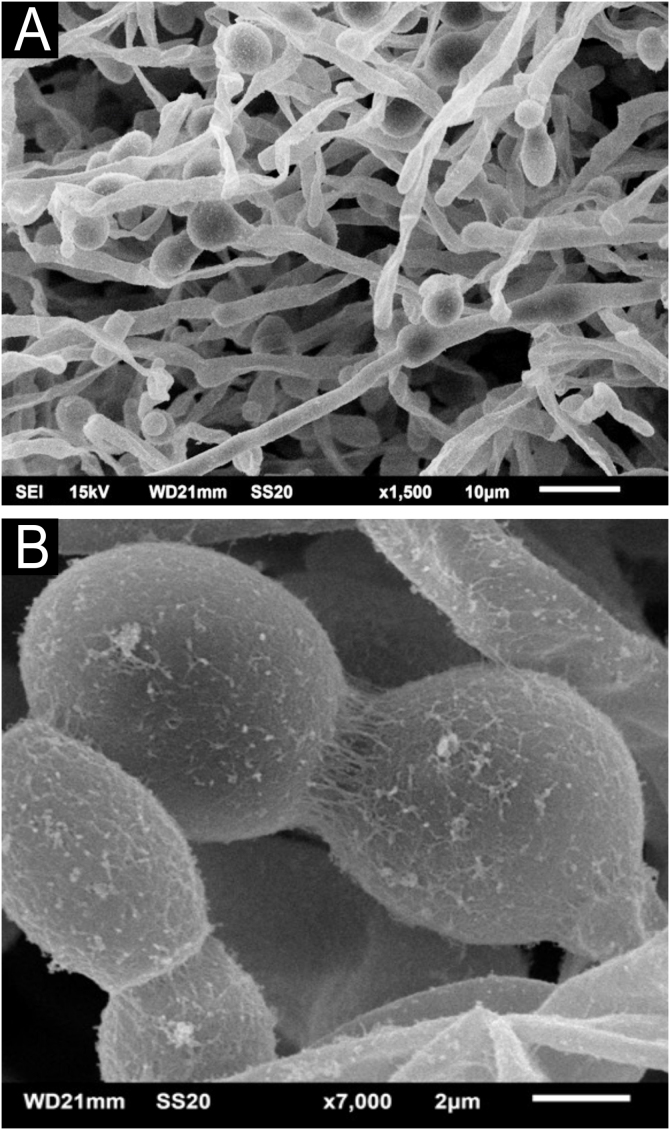
SEM of *T. mucoides* colony – (A) Fungal filaments and blastoconidia not adhered by extracellular matrix (×1,500). (B) Detail of blastoconidia with discrete extracellular matrix production (×7.000).

The SEM examination of the *T. mucoides* colony obtained from the mycotheque shows filaments and blastoconidia, without the formation of the evident extracellular matrix adhering the fungal cells to each other (Fig. 9A), as seen in the colony obtained in the present case of white piedra. At high magnifications, it was possible to observe a small production of a fibrillar substance between the blastoconidia (Fig. 9B).

## Discussion

The findings from the examination of the piedra nodule in this report are similar to previously published findings regarding its ultrastructure, with hyphae (in sections with transmission electron microscopy analysis) and spores surrounded by a cementing substance on the surface, in the SEM analysis.^11^

Regarding the examination of the colony obtained from the index case presented herein, a significant formation of extracellular matrix was observed, adhering to and covering the fungal structures, forming a biofilm. This matrix must correspond to the cementing substance described in the condition.

Biofilm formation was first demonstrated in the 1970s and is characterized by an extracellular matrix with embedded etiological agents.^12^, ^13^ It must have the function of protecting the etiological agents against ultraviolet radiation, extreme temperatures and pH, salinity and pressure, and may also be involved in resistance to antibiotics and antifungals.^12^ It may be formed by polysaccharides, proteins, or fats.^12^

Several fungi of dermatological interest have been described as biofilm formers, such as *T. rubrum* and T. mentagrophytes, and in the genera *Aspergillus*, *Candida* and *Cryptococcus*.^14^, ^15^, ^16^

Biofilm formation has also been reported in relation to the genus *Trichosporon*,^17^, ^18^, ^19^, ^20^ and interspecies variation has been found, which can be classified as weak, medium or strong biofilm producers.^17^ In a publication with a series of 53 strains of *Trichosporon* obtained from urinary samples, around 10% were medium biofilm producers and the majority were weak producers.^19^ With *T. asahii* obtained from blood culture, it was experimentally demonstrated that the fungus adheres to culture plates and begins to produce biofilm within a few hours.^20^

An experimental investigation has demonstrated that several species of *Trichosporon* can adhere to hair, forming piedra.^21^

Possibly, strains with low production of extracellular matrix may not form the piedra nodule, as this would be necessary to form the hair concretion. Similar to what was demonstrated here, with the intense production of extracellular matrix by the colony obtained from affected hair, in contrast to the low production of extracellular matrix by *T. mucoides* obtained from the mycotheque, it is also possible that this strain kept frozen in the mycotheque may have modified the likelihood of producing the extracellular matrix.

The formation of the cementing substance firmly adhered to the hair and of the biofilm could be responsible for the slow response to the topical or systemic antifungal therapy in white piedra.

It is ​​suggested that extracellular matrix synthesis plays a crucial role in the firm adhesion of fungal cells to each other, resulting in the formation of piedra nodules, as it has also been demonstrated with the etiological agent of black piedra.^22^ These SEM findings should be expanded to include more species of *Trichosporon*, whether piedra-forming or not.

## Financial support

None declared.

## Authors' contributions

Hiram Larangeira de Almeida Jr: Approval of the final version of the manuscript; design and planning of the study; drafting and editing of the manuscript; collection, analysis and interpretation of data; effective participation in research orientation; intellectual participation in the propaedeutic and/or therapeutic conduct of the studied cases, critical review of the literature; critical review of the manuscript.

Thales de Moura Assis: Approval of the final version of the manuscript; design and planning of the study; drafting and editing of the manuscript; collection, analysis and interpretation of data; intellectual participation in the propaedeutic and/or therapeutic conduct of the studied cases; critical review of the literature; critical review of the manuscript.

Eduardo Camargo Faria: Approval of the final version of the manuscript; design and planning of the study; drafting and editing of the manuscript; collection, analysis and interpretation of data; intellectual participation in the propaedeutic and/or therapeutic conduct of the studied cases; critical review of the literature; critical review of the manuscript.

Viviane Mazo Fávero Gimenes: Approval of the final version of the manuscript; drafting and editing of the manuscript; collection, analysis and interpretation of data; intellectual participation in the propaedeutic and/or therapeutic conduct of the studied cases; critical review of the literature; critical review of the manuscript.

## Conflicts of interest

None declared.
